# Improving the meiotic competence of small antral follicle-derived porcine oocytes by using dibutyryl-cAMP and melatonin

**DOI:** 10.5713/ab.23.0371

**Published:** 2024-02-23

**Authors:** Jakree Jitjumnong, Pin-Chi Tang

**Affiliations:** 1 Department of Animal Science, National Chung Hsing University, 40227 Taichung, Taiwan; 2 The iEGG and Animal Biotechnology Center, National Chung Hsing University, 40227 Taichung, Taiwan

**Keywords:** Cumulus Cell Expansion, Dibutyryl-cAMP, Embryo Development, Human Chorionic Gonadotropin, Melatonin, Small Antral Follicle

## Abstract

**Objective:**

We increased the nuclear maturation rate of antral follicle derived oocytes by using a pre-*in vitro* maturation (IVM) culture system and improved the developmental potential of these porcine pathenotes by supplementing with melatonin. Furthermore, we investigated the expression patterns of genes involved in cumulus expansion (*HAS2*, *PTGS2*, *TNFAIP6*, and *PTX3*) derived from small and medium antral follicles before and after oocyte maturation.

**Methods:**

Only the cumulus oocyte-complexes (COCs) derived from small antral follicles were induced with [Pre-SF(+)hCG] or without [Pre-SF(-)hCG] the addition of human chorionic gonadotropin (hCG) during the last 7 h of the pre-IVM period before undergoing the regular culture system. The mature oocytes were investigated on embryonic development after parthenogenetic activation (PA). Melatonin (10^−7^ M) was supplemented during *in vitro* culture (IVC) to improve the developmental potential of these porcine pathenotes.

**Results:**

A pre-IVM culture system with hCG added during the last 7 h of the pre-IVM period [Pre-SF(+)hCG] effectively supported small antral follicle-derived oocytes and increased their nuclear maturation rate. The oocytes derived from medium antral follicles exhibited the highest nuclear maturation rate in a regular culture system. Compared with oocytes cultured in a regular culture system, those cultured in the pre-IVM culture system exhibited considerable overexpression of *HAS2*, *PTGS2*, and *TNFAIP6*. Porcine embryos treated with melatonin during IVC exhibited markedly improved quality and developmental competence after PA. Notably, melatonin supplementation during the IVM period can reduce and increase the levels of intracellular reactive oxygen species (ROS) and glutathione (GSH), respectively.

**Conclusion:**

Our findings indicate that the Pre-SF(+)hCG culture system increases the nuclear maturation rate of small antral follicle-derived oocytes and the expression of genes involved in cumulus expansion. Melatonin supplementation during IVC may improve the quality and increase the blastocyst formation rate of porcine embryos. In addition, it can reduce and increase the levels of ROS and GSH, respectively, in mature oocytes, thus affecting subsequent embryos.

## INTRODUCTION

The developmental competence of oocytes to progress through meiosis can be determined on the basis of their diameter, follicle size, and cumulus cell layers. When oocytes are separated from small antral follicles, approximately 50% of the cumulus oocyte-complexes (COCs) have less cumulus cell layers [1]. Oocytes derived from medium and large antral follicles exhibit higher developmental competence in terms of reaching the metaphase-II (MII) and blastocyst stages than do those derived from small antral follicles [2]. Oocytes have been classified as those derived from medium antral follicles (diameter, 3 to 6 mm) and those derived from small antral follicles (diameter, <3 mm) on the basis of their follicle size. Regular *in vitro* maturation (IVM) approaches may fail to adequately foster the development of oocytes derived from small antral follicles [3]; even the approaches that facilitate the progression of oocytes to the MII stage lead to unsatisfactory cytoplasmic maturation, resulting in low developmental competence in these oocytes. This is a concern because small antral follicles constitute 71.8% of the surface of prepubertal ovaries before they are discarded [4]. Therefore, to avoid compromising resources such as small antral follicles, simulated physiological oocyte maturation (SPOM) has been proposed to improve the developmental competence and increase the nuclear maturation rate of oocytes derived from small antral follicles [5].

SPOM is an IVM approach that improves oocyte capacitation through cyclic adenosine 3′5′-monophosphate (cAMP) [5]. Oocytes with meiotic competence normally contain all proteins required for meiosis and survival during embryonic development under *in vivo* conditions by the end of folliculogenesis [6]. However, as follicle generated cAMP and move through the gap junctions between cumulus cells and oocytes, the cAMP level in oocytes decrease when they are isolated from follicles. cAMP activates protein kinase A (PKA) to prevent germinal vesicle breakdown (GVBD); therefore, the downregulation of cAMP expression facilitates GVBD. The inactive form of PKA induces meiosis resumption and activates maturation-promoting factor (MPF) in oocytes. SPOM helps resolve this problem by supplementing oocytes with cAMP modulators during the pre-IVM period [7]. This supplementation prevents spontaneous meiosis and nuclear and cytoplasmic maturation during pre-IVM, resulting in oocyte synchronization during IVM, which improves oocyte efficiency [8].

In the present study, dibutyryl-cAMP (dbcAMP; a cAMP analog) was used to increase cAMP levels, inhibit MPF activity, and delay the meiosis stage in porcine oocytes [9]. After the synchronization of meiotic maturation, the levels of intracellular cAMP were reduced by washing, which was followed by the transfer of cells to a regular culture system to promote oocyte maturation [5]. dbcAMP can activate its receptors in cumulus cells and oocytes, thus improving the meiotic competence and cytoplasmic maturation of oocytes derived from small antral follicles.

For optimal IVM, porcine oocytes generally require 44 h to progress to the MII stage. However, this culturing duration may be insufficient for poor-quality oocytes (less layers of cumulus cells) to achieve full meiotic competence. Compared with a period of 44 h, a more extended IVM period (44 to 52 h) may lead to an increased nuclear maturation rate of poor-quality oocytes [1] but not an increased formation rate of blastocysts. Furthermore, the intracellular levels of reactive oxygen species (ROS) and glutathione (GSH) are higher and lower, respectively, in oocytes cultured for 52 h than in those cultured for 44 h. Increased ROS and reduced GSH levels indicate defective embryonic development [10]. We hypothesized that culturing oocytes in a pre-IVM culture system and, subsequently, in a regular culture system would improve their developmental competence and increase intracellular ROS levels. We further hypothesized that reducing and increasing intracellular ROS and GSH levels during IVM or *in vitro* culture (IVC) would improve the quality and developmental competence of oocytes. The addition of antioxidants to culture media during IVM and IVC may decrease ROS levels and increase GSH levels; it may also increase the developmental competence of porcine embryos [11].

Melatonin (N-acetyl-5-methoxytryptamine) is a natural hormone and the principal secretory product of the mammalian pineal gland. This hormone is essential for the regulation of physiological processes, including sleep, circadian rhythm, seasonal reproduction, and steroidogenesis [10]. Melatonin exhibits free radical–scavenging, antioxidative, and antiapoptotic activities. Thus, it can directly scavenge toxic oxygen derivatives and reduce intracellular ROS levels. Melatonin can also induce the activity of antioxidative enzymes, such as GSH and superoxide dismutase. Moreover, melatonin supplementation during IVM decreases ROS levels in porcine oocytes and markedly increases the blastocyst formation rate [12]. During IVM, melatonin protects porcine oocytes from heat stress by reducing ROS levels and apoptosis while increasing intracellular GSH levels [13]. Therefore, the aims of this study were to increase the nuclear maturation rate of small antral follicle-derived oocytes by culturing them in a pre-IVM culture system and explore the gene expression patterns of hyaluronan synthase 2 (*HAS2*), prostaglandin-endoperoxide synthase 2 (*PTGS2*), tumor necrosis factor–stimulated gene 6 (*TNFAIP6*), and pentraxin 3 (*PTX3*) in cumulus cells derived from small and medium antral follicles before and after the maturation of oocytes either in regular or pre-IVM culture systems. To improve the quality and development of blastocyst formation, we supplemented IVC and IVM media with melatonin and determined the outcomes of developmental competence of oocytes.

## MATERIALS AND METHODS

### Chemicals

All reagents and chemicals used in this study were purchased from Sigma-Aldrich (St. Louis, MO, USA) unless indicated otherwise.

### Oocyte recovery and classification

**Figure 1:**
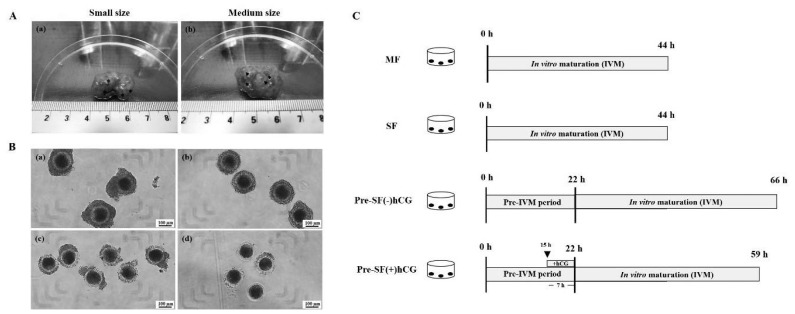
(A) Pig ovaries derived from (a) small (diameter, <3 mm; asterisk) and (b) medium (diameter, 3 to 5 mm; arrowhead) antral follicles. (B) Grades of COCs harvested from pig ovaries. (a) Grade I oocytes exhibit an uniform appearance with multiple cumulus cell layers and cytoplasm. (b) Grade II oocytes exhibit a uniform and intact corona radiata surrounded by another layer of cumulus cells and cytoplasm; however, the number of cumulus cell layers is less than that of cumulus oophorus cell layers. (c) Grade III oocytes lack uniformity, as indicated by the mosaic transparency of the cytoplasm; incomplete corona radiata and partially denuded oocytes are noted. (d) Grade IV oocytes exhibit high mosaic transparency and cytoplasm fragmentation; sparse corona radiata and cumulus oophorus cells. Scale bar, 100 μm. (C) *In vitro* cultivation of porcine oocytes. COCs derived from small and medium antral follicles were cultured for 44 h in regular culture systems (MF and SF). By contrast, COCs derived only from small antral follicles were cultured for 22 h in pre-IVM medium containing 1 mM dibutyryl-cAMP and then in standard IVM medium for 44 h [Pre-SF(−)hCG] or standard IVM medium with hCG for 37 h [Pre-SF(+)hCG]. COC, cumulus–oocyte complex; IVM, *in vitro* maturation; and hCG, human chorionic gonadotropin.

Porcine ovaries were obtained from prepubertal gilts at a local abattoir. The specimens were transported to our laboratory in phosphate-buffered saline (PBS; 30°C to 35°C) containing 1% penicillin and streptomycin (Invitrogen Corporation, Carlsbad, CA, USA) within 2 h of collection. The ovaries were washed thrice with PBS at 37°C (Figure 1). An 18-G needle attached to a disposable 10-mL syringe was used to aspirate COCs on the basis of the follicular diameters of medium (3 to 5 mm) and small (<3 mm) antral follicles. Aspirated COCs with at least three layers of compact cumulus cells and homogenous cytoplasm were carefully selected under stereomicroscopy and washed thrice with HEPES medium before culture (Figure 1B).

### Regular and pre-*in vitro* maturation culture systems

COCs derived from medium and small antral follicles, i.e. MF and SF, respectively, were cultured in regular culture systems [regular medium; medium-199 supplemented with 10% fetal bovine serum (FBS), 10% porcine follicular fluid (pFF), 0.1 mg/mL of sodium pyruvate, 0.1 IU/mL of porcine follicle-stimulating hormone (pFSH), 10 IU/mL of human chorionic gonadotropin (hCG), and 1 μg/mL of estradiol-17β] under 5% CO_2_ at 39°C for 44 h. In the pre-IVM culture system (pre-IVM medium; αMEM supplemented with 5% FBS, 5% pFF, 0.1 mg/mL of sodium pyruvate, 1.8 μM estradiol-17β, 0.01 IU/mL of pFSH, and 1 mM dbcAMP), only the COCs aspirated from small antral follicles were cultured with or without the addition of hCG during the last 7 h of the pre-IVM period (15 to 22 h; Pre-SF[−]hCG and Pre-SF[+]hCG, respectively) under 5% CO_2_ at 39°C for 22 h [14]. Subsequently, the COCs cultured in both the Pre-SF(−)hCG and Pre-SF(+)hCG culture systems were washed thrice with the regular medium. Then, the washed cells, which were cultured in the Pre-SF(−)hCG and Pre-SF(+)hCG culture systems, were cultured in regular medium for 44 and 37 h, respectively (Figure 1C). Using a syringe attached to an 18-G needle, pFF was aspirated from the follicles (diameter, 3 to 6 mm) into a 15-mL tube for pFF preparation. To remove the blood, the collected pFF was centrifuged twice at 3,500×*g* for 30 min at 4°C and filtered through a 0.45 and 0.22 μm syringe filter (Pall Corporation, Port Washington, NY, USA). Next, the supernatant was aliquoted into 1.5-mL microcentrifuge tubes and maintained at −20°C until needed. All media, including the IVC medium, were equilibrated at 39°C under 5% CO_2_ for at least 1 h in regular and pre-IVM media and for 4 h in the IVC medium.

### Gene expression analysis

Before and after IVM, total RNA was isolated from cumulus cells, which were isolated from 50 oocytes by gently pipetting with 0.1% hyaluronidase, by using RNeasy Micro Kit (Qiagen, Hilden, Germany) according to the manufacturer’s instructions. The obtained RNA was suspended into 20 μL of RNase-free water and reverse-transcribed into cDNA by using Transcriptor First Strand cDNA Synthesis Kit (Roche, Mannheim, Germany) according to the manufacturer’s instructions. Through quantitative polymerase chain reaction (PCR), the expression patterns of genes involved in cumulus expansion (*HAS2*, *PTGS2*, *TNFAIP6*, and *PTX3*) were analyzed using a StepOne Real-Time PCR System (Applied Biosystems; Thermo Fisher Scientific, Carlsbad, CA, USA). The mRNA levels of *HAS2*, *PTGS2*, *TNFAIP6*, and *PTX3* were measured in cumulus cells. The expression level of each target gene in the cumulus cells was quantified on the basis of the abundance of corresponding mRNA relative to that of glyceraldehyde 3-phosphate dehydrogenase (*GAPDH*) mRNA. Relative quantification was performed by comparing the genes in terms of threshold cycle (C_t_); then, the relative mRNA level was calculated on the basis of *GAPDH* level in the cumulus cells. The gene-specific primers are listed in Table 1.

### Parthenogenetic activation of oocytes

After maturation, the oocytes were denuded of cumulus cells by being aspirated for 2 to 3 min in a washing medium containing 0.1% hyaluronidase. Before activation, the denuded oocytes were washed thrice in Dulbecco’s PBS (DPBS; HyClone, Logan, UT, USA). Then, the denuded oocytes were activated through incubation with 5 μM Ca^2+^ ionomycin (Sigma-Aldrich, Merck KGaA, Darmstadt, Germany) in DPBS for 5 min. The activated oocytes were washed thrice in porcine zygote medium 5 (PZM-5; Funakoshi Corporation, Tokyo, Japan) and then incubated in PZM-5 medium containing 2 mM 6-dimethylaminopurine (6-DMAP) for 5 h at 39°C under 5% CO_2_.

### *In vitro* embryo culture and evaluation

Presumptive zygotes resulting from parthenogenetic activation (PA) in the presence of 6-DMAP were removed after 5 h of incubation by washing the oocytes with DPBS. For embryo development, the activated oocytes were cultured in IVC medium containing 50-μL microdrops (10 to 15 embryos/drop) of PZM-5 supplemented with 0.4% (w/v) bovine serum albumin (BSA). The IVC medium was covered in paraffin oil and incubated for 7 days at 39°C under 5% CO_2_ in a humidified atmosphere. The medium was changed on Days 2 (48 h after PA) and 4 (96 h after PA). For the analysis of the embryos, the day on which the newly formed presumptive zygotes were transferred to the IVC medium was set as Day 0. On Day 2, the cleavage rate of the cultivated embryos was calculated under a stereomicroscope. For melatonin treatment, melatonin powder was dissolved in ethanol (stock concentration, 10^−5^ M) and added to the IVC medium (final concentration, 10^−7^ M). The control groups received the same amount of ethanol. On Day 7, after PA, the blastocysts were removed and fixed for 1 h in 4% formaldehyde containing 0.1% polyvinyl alcohol (PVA)–DPBS. The fixed blastocysts were washed thrice before being stained with Hoechst 33342 (10 μg/mL; incubation conditions: 10 min at room temperature in the dark). After staining, the blastocysts were gently placed on a glass slide into a drop of antifade medium and were covered with a coverslip. Then, the slides were sealed with nail polish and observed under a fluorescence microscope (Nikon, Japan) at 200× magnification. All experiments were repeated four times.

### Measurement of intracellular reactive oxygen species and glutathione levels

To measure the levels of intracellular ROS and GSH, denuded MII-stage oocytes were washed with 0.1% hyaluronidase after 44-h culture in the MF and 59-h culture in the Pre-SF(+)hCG culture systems in the presence or absence of melatonin. In brief, 2′,7′-dichlorodihydroflurscein diacetate (H_2_DCFDA; green fluorescence; Invitrogen, USA) and 4-chloromethyl-6.8-difluoro-7-hydroxycoumarin (CMF_2_HC; blue fluorescence; Invitrogen, USA) were used to evaluate ROS and GSH levels, respectively, in oocyte cytoplasm. Mature oocytes were washed thrice with 0.1% PVA–DPBS and incubated with 10 μM H_2_DCFDA or CMF_2_HC at 37°C in the dark for 30 min. Thereafter, the stained oocytes were washed thrice, added into 50 μL of 0.1% PVA–DPBS, and observed under a fluorescence microscope (Olympus, Tokyo, Japan; ROS, 460 nm; GSH, 370 nm). The obtained fluorescence images were analyzed using ImageJ (version 1.46r) to determine the fluorescence intensity of the oocytes derived from Pre-SF(+)hCG and Pre-SF(+)hCG + melatonin compared with that of the oocytes cultured in the MF system (positive control) after background adjustment. The independent experiment was repeated four times (n≥25 per group). The relative fluorescence intensities were considered to indicate the fluorescence intensities of the ROS and GSH.

### Statistical analysis

The Student’s t test was used to compare the expression patterns of genes before and after oocyte maturation in the regular and pre-IVM culture systems. Data regarding the relative abundance of mRNA is presented as the mean±standard error of mean. Chi-square analysis was performed to compare the percentage data regarding the maturation rate, cleavage rate, and blastocyst formation rate. The means of total cell number of blastocysts and intracellular ROS and GSH levels among treatments were compared using Duncan’s new multiple range tests. All of the analysis was performed by SAS (SAS Institute, Cary, NC, USA). Statistical significance was set at p<0.05. A value of 0.05≤p<0.10 was considered to indicate a tendency [15].

## RESULTS

### Comparison of the regular and pre-IVM culture systems in terms of cumulus expansion and nuclear maturation rate

**Figure 2:**
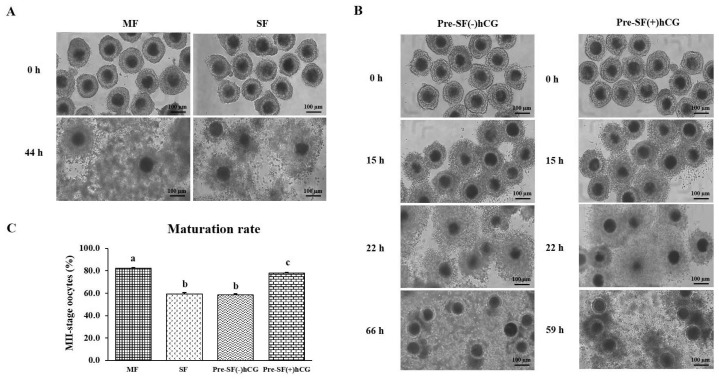
Morphological changes in COCs and the effects of different culture systems on the nuclear maturation rate. (A) Oocytes derived from small (SF) and medium (MF) antral follicles were cultured in standard IVM medium for 44 h in COCs; the only difference was in the use of COCs derived from different-sized follicles. Scale bar, 100 μm. (B) COCs derived from small antral follicles (diameter <3 mm) were cultured in pre-IVM medium containing 1 mM dibutyryl-cAMP for 22 h and then in standard IVM medium for 44 h [Pre-SF(−)hCG] or in standard IVM medium with hCG for 37 h [Pre-SF(+)hCG]. Scale bar, 100 μm. (C) Effects of different culture systems on the meiotic competence of the oocytes derived from small and medium antral follicles. The experiment was repeated 10 times. The data are presented in terms of mean±standard error of the mean values. COC, cumulus–oocyte complex; IVM, *in vitro* maturation; hCG, human chorionic gonadotropin. ^a–c^ Different letters above the bars indicate significant differences (p<0.05).

As shown in Figure 2, when cultured in a regular culture system, oocytes derived from MF had a significantly higher (p<0.05) nuclear maturation rate (82.14%; n = 1,426) than did those derived from SF (Figure 2C). Oocytes cultured in Pre-SF(+)hCG had a lower nuclear maturation rate than did those cultured in MF; however, oocytes cultured in Pre-SF(+)hCG had a significantly higher (p<0.05) nuclear maturation rate than did oocytes cultured in SF and Pre-SF(−)hCG [77.80% (n = 1101), 59.11% (n = 1209), and 58.62% (n = 1,411), respectively] culture systems (Figure 2C). No significant difference (p>0.05) was identified between the oocytes cultured in SF and those cultured in Pre-SF(−)hCG in terms of nuclear maturation rate (Figure 2C). We further explored cumulus expansion (Figure 2A and 2B). Before oocyte IVM, COCs were isolated from different-sized follicles and cultured in regular and pre-IVM culture systems. After 44 h of culture in a regular culture system, the cumulus cells in COCs derived from medium antral follicles exhibited full complement and expansion than did those derived from small antral follicles (Figure 2A). After 15 h of culture in the pre-IVM culture system (both systems), the COCs derived from small antral follicles exhibited cumulus cell proliferation (Figure 2B). However, unlike the COCs cultured in Pre-SF(−)hCG, the COCs cultured in Pre-SF(+)hCG exhibited a full expansion of cumulus cells (Figure 2B). Thus, Pre-SF(+)hCG appears to be a complete culture system suitable for supporting small antral follicle-derived oocytes [1]. Therefore, the oocytes that were derived from medium and small antral follicles and cultured in regular (MF) and Pre-SF(+)hCG culture systems were analyzed further.

### Expression patterns of cumulus expansion–related genes before and after oocyte maturation

**Figure 3:**
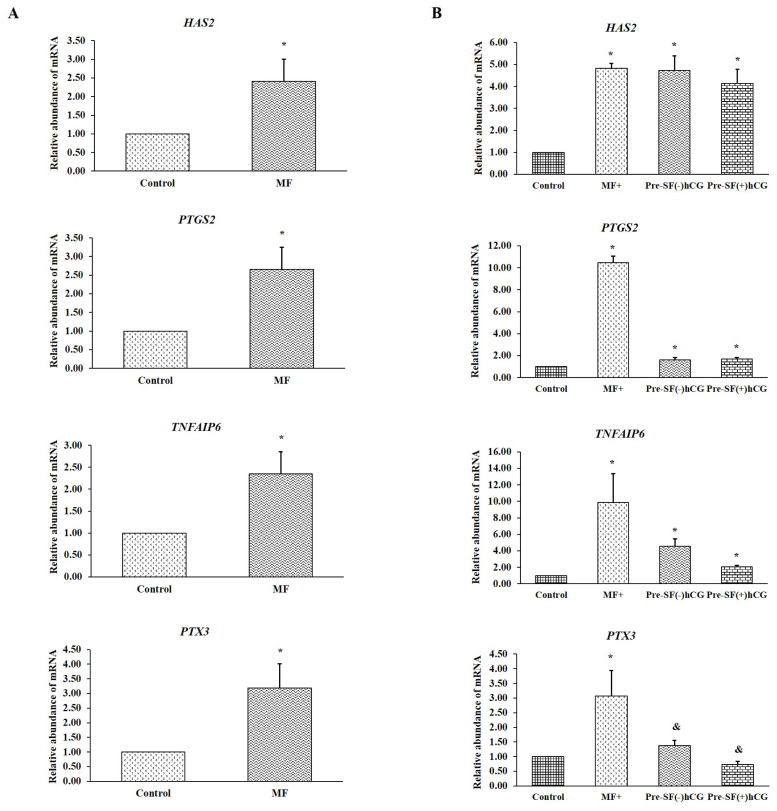
Gene expression in cumulus cells before (A) and after (B) *in vitro* maturation in different culture systems. Cumulus cells derived from small antral follicles were used as control cells. Total RNA was extracted from oocytes and reverse-transcribed. Quantitative polymerase chain reaction was performed. The relative expression patterns of genes involved in cumulus expansion (*HAS2*, *PTGS2*, *TNFAIP6*, and *PTX3*) were explored. The expression of glyceraldehyde 3-phosphate dehydrogenase was analyzed to normalize the data. The data are presented in terms of mean±standard error of the mean values. Three independent experiments were performed for each culture system. *HAS2*, hyaluronan synthase 2; *PTGS2*, prostaglandin-endoperoxide synthase 2; *TNFAIP6*, tumor necrosis factor–stimulated gene 6; *PTX3*, pentraxin 3. Asterisks above the bars indicate significant differences from the control group (p<0.05).

Real-time PCR was performed to evaluate the expression levels of genes involved in cumulus expansion (*HAS2*, *PTGS2*, *TNFAIP6*, and *PTX3*) before and after oocyte maturation. Cumulus cells derived from small antral follicles were used as a control. Before culture, the cumulus cells derived from medium-sized follicles had a considerably higher relative abundance of mRNA (for all four genes) than did the oocytes derived from small antral follicles (p<0.05; Figure 3A). The oocytes derived from medium antral follicles and cultured in regular medium (MF) had significantly higher (p<0.05) mRNA levels for all four genes during cumulus expansion than did those derived from small antral follicles and cultured in regular medium (SF; Figure 3B). Notably, significant increases (p<0.05) were noted in the mRNA levels of *HAS2*, *PTGS2*, and *TNFAIP6* in the oocytes derived from small antral follicles and cultured in Pre-SF(−)hCG and Pre-SF(+)hCG. By contrast, the increase in the mRNA level of *PTX3* in oocytes cultured in these pre-IVM culture systems compared with that in oocytes cultured in a regular culture system (SF) exhibited a tendency toward significance (p = 0.08 and 0.06, respectively; Figure 3B). These findings indicate that oocytes derived from medium antral follicles respond well to regular culture systems (MF). Compared with the regular (SF) and Pre-SF(−)hCG culture systems, the Pre-SF(+)hCG culture system considerably improved the nuclear maturation rate in the oocytes derived from small antral follicles. Furthermore, MF and Pre-SF(+)hCG, which ensured a high nuclear maturation rate, appeared to be essential for promoting the transcription of the genes involved in cumulus expansion.

### Effects of the regular and pre-IVM culture systems on the quality and developmental competence of oocytes derived from small and medium antral follicles

**Figure 4:**
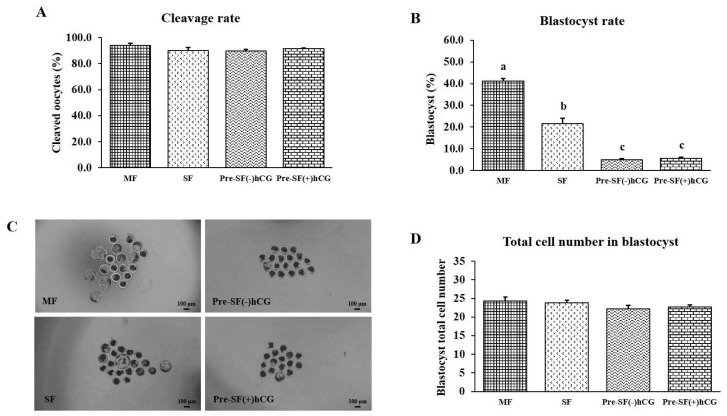
Effects of regular and pre-IVM + dibutyryl-cAMP culture systems on the developmental competence of the oocytes derived from medium and small antral follicles. (A) Cleavage rate. (B) Blastocyst formation rate. (C) Blastocyst morphology. Scale bar, 100 μm. (D) The total number of blastocyst cells was counted after differential staining with Hoechst 33342. All experiments were repeated four times. IVM, *in vitro* maturation. ^a–c^ Different letters above the bars indicate significant differences (p<0.05).

Mature oocytes (n = 697) derived from different-sized follicles (four independent replicates) were used to investigate the effects of the regular (MF [n = 152] and SF [n = 170]) and pre-IVM [Pre-SF(−)hCG (n = 166) and Pre-SF(+)hCG (n = 209)] culture systems on embryonic development after PA. No significant differences (p>0.05) were observed in the cleavage rates and total cell numbers for the regular and pre-IVM culture systems (Figure 4A and 4D). In the regular culture system, the developmental competence of the oocytes derived from medium antral follicles was higher (p<0.05) in terms of the blastocyst formation rate on Day 7 than that of the oocytes derived from small antral follicles (blastocyst formation rate: 41.17% vs 21.61%; Figure 4B and 4C). Regarding the oocytes derived from small antral follicles, those cultured in a regular culture (SF) system had a significantly higher (p<0.05) blastocyst formation rate than did those cultured in the Pre-SF(–)hCG and Pre-SF(+)hCG culture systems (blastocyst formation rate: 21.61%, 4.85%, and 5.59%, respectively). By contrast, no difference (p>0.05) was observed between the Pre-SF(–)hCG and Pre-SF(+)hCG (Figure 4B and 4C).

### Effects of melatonin supplementation on the quality and developmental competence of porcine oocytes

**Figure 5:**
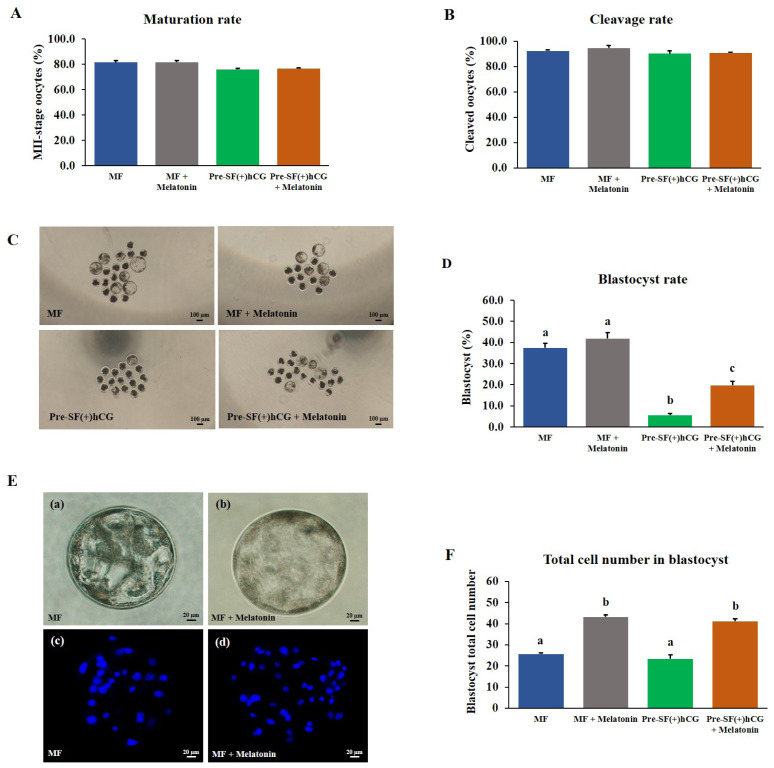
Effects of melatonin supplementation on porcine embryonic development. (A) Nuclear maturation rate. (B) Cleavage rate. (C) Blastocyst morphology. Scale bar, 100 μm. (D) Blastocyst formation rate. (E) Porcine parthenogenetic blastocysts and cells cultured in the absence (a,c) and presence (b,d) of melatonin; staining was performed using Hoechst 33342. Blue spots indicate cell nuclei within blastocysts. Scale bar, 20 μm. (F) Total number of blastocyst cells. All experiments were repeated four times. ^a–c^ Different letters above the bars indicate significant differences (p<0.05).

The oocytes derived from medium and small antral follicles and cultured in regular (MF) and Pre-SF(+)hCG culture systems had a high nuclear maturation rate (Figure 5). Thus, these culture systems were selected to assess the effects of melatonin supplementation. A study reported that a melatonin concentration of 10−7 M markedly increased the blastocyst formation rate during IVC in porcine embryos prepared through *in vitro* fertilization [16]. The COCs derived from medium and small antral follicles were cultured in regular (44 h) and pre-IVM (59 h) culture systems, respectively. No significant difference (p>0.05) was noted between the culture systems in terms of the nuclear maturation rate of the oocytes (Figure 5A). Similarly, 2 days after PA, no significant difference (p>0.05) was noted between the culture systems in terms of the cleavage rate (Figure 5B). However, the oocytes cultured in the Pre-SF(+)hCG + melatonin culture system had a significantly higher (p<0.05) blastocyst formation rate on Day 7 than did those cultured in the Pre-SF(+)hCG (blastocyst formation rate: 19.69% vs 5.57%); no difference was observed between the MF and MF + melatonin culture systems in terms of the blastocyst formation rate (Figure 5C and D). The total number of blastocyst cells was significantly higher in the MF + melatonin and Pre-SF(+)hCG + melatonin than in the MF and Pre-SF(+)hCG (total cell number: 43.18, 41.10, 25.63, and 23.33, respectively; Figure 5E and 5F). Although no significant difference (p>0.05) was observed between the MF and MF + melatonin in terms of the blastocyst formation rate, the MF + melatonin was determined to be a better culture system than the MF. These findings suggest that melatonin supplementation during IVC can increase the blastocyst formation rate, particularly in oocytes derived from small antral follicles. Considering the positive effects of melatonin, we performed subsequent experiments using oocytes cultured in the presence of melatonin.

### Effects of melatonin supplementation on the levels of intracellular ROS and GSH in mature oocytes

**Figure 6:**
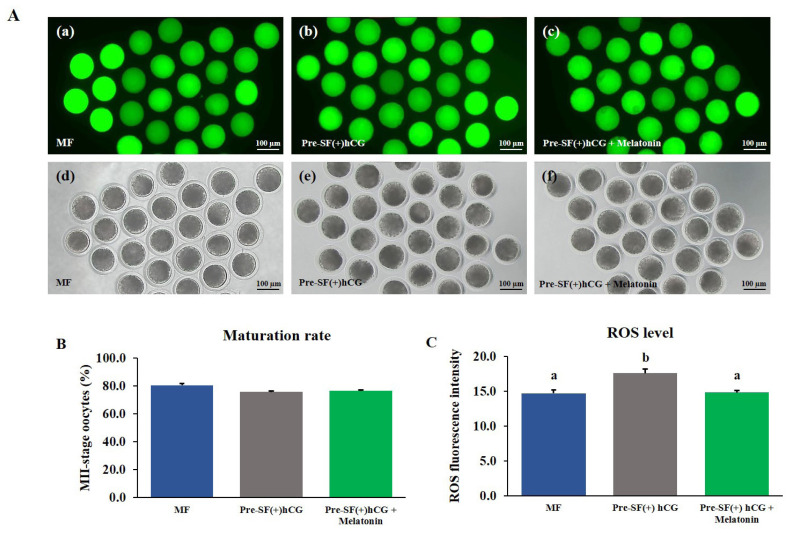
Effects of melatonin supplementation on the maturation and intracellular ROS levels of mature oocytes. (A) Fluorescence (a–c) and bright-field (d–f) images of oocytes treated with 2′,7′-dichlorodihydroflurscein diacetate to measure intracellular ROS levels. The oocytes were cultured in regular MF, Pre-SF(+)hCG, and Pre-SF(+)hCG + melatonin. Scale bar, 100 μm. (B) Nuclear maturation rate. (C) Fluorescence intensity. All experiments were repeated four times. ROS, reactive oxygen species; hCG, human chorionic gonadotropin. ^a,b^ Different letters above the bars indicate significant differences (p<0.05).

**Figure 7:**
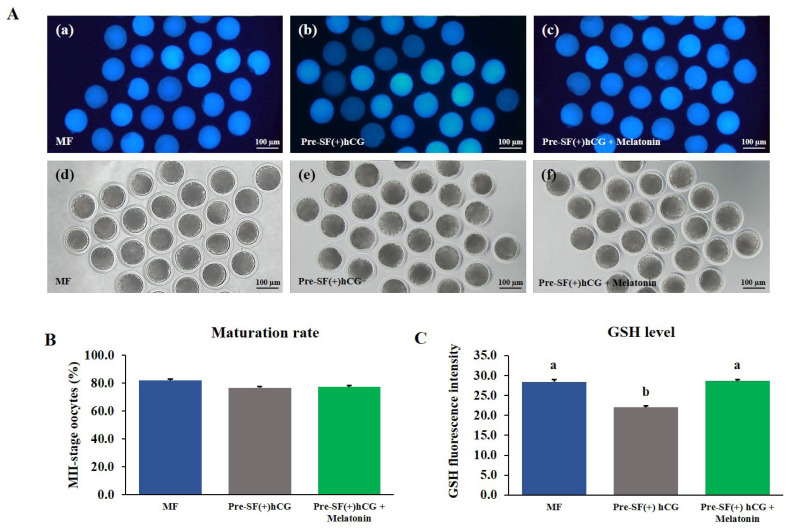
Effects of melatonin supplementation on the maturation and intracellular GSH levels of mature oocytes. (A) Fluorescence (a–c) and bright-field (d–f) images of oocytes treated with 4-chloromethyl-6.8-difluoro-7-hydroxycoumarin to measure intracellular GSH levels. The oocytes were cultured in regular MF, Pre-SF(+)hCG, and Pre-SF(+)hCG + melatonin. Scale bar, 100 μm. (B) Nuclear maturation rate. (C) Fluorescence intensity. All experiments were repeated four times. GSH, glutathione; hCG, human chorionic gonadotropin. ^a,b^ Different letters above the bars indicate significant differences (p<0.05).

The COCs derived from medium antral follicles and cultured in a regular culture system (MF) for 44 h without melatonin supplementation were used as a positive control. The COCs derived from small antral follicles were cultured in the pre-IVM culture system for 59 h with or without melatonin supplementation during IVM. Melatonin did not significantly (p>0.05) increase the nuclear maturation rate of the oocytes (Figure 6B and 7B). However, in the oocytes cultured in the Pre-SF(+)hCG + melatonin, the level of intracellular ROS decreased significantly (p>0.05; Figure 6A and 6C) after maturation, whereas that of GSH increased significantly (p>0.05; Figure 7A and 7C). The changes in the levels of intracellular ROS and GSH were similar between the oocytes cultured in the Pre-SF(+)hCG + melatonin culture system and the positive control.

## DISCUSSION

We investigated the developmental competence of oocytes derived from small and medium antral follicles in terms of their progression to the MII stage and rate of blastocyst formation. Only oocytes derived from small antral follicles were cultured in pre-IVM culture systems to increase the nuclear maturation rate. On the basis of other studies, porcine oocytes derived from small antral follicles (diameter, 1 to 3 mm) were cultured in an oocyte growth medium for 24 h. Then, the cells were transferred to an oocyte maturation medium and cultured for 20 h. By contrast, some oocytes were directly cultured in oocyte maturation medium. After 44 h, only 36% and 55% of the oocytes cultured in the aforementioned two-media and direct culture systems reached the MII stage, respectively. By contrast, in a relevant study, approximately 80% of oocytes derived from large antral follicles (diameter, 4 to 6 mm) reached this stage. It is proposed that the porcine oocytes from large follicles had a considerably greater amount of chromatin surrounding the nucleolus, in which the transcription level was low and might be enhanced in the degree of meiotic progression and developmental capacity [17]. Furthermore, porcine oocytes derived from small antral follicles have a low capacity for estradiol-17β production during IVM and can further suppress progesterone production at the end of the IVM period; however, oocytes derived from medium antral follicles produce high levels of these hormones [18]. In this study, our finding revealed a consistent trend as described previously. The oocyte obtained from small antral follicles and undergone the process of pre-maturation would have similar capacity to reach to the MII stage as those collected from large antral follicles undergone the regular IVM process. Moreover, the small antral follicle-derived oocytes cultured in Pre-SF(+)hCG had a higher nuclear maturation rate than did those cultured in regular medium (44 h) and Pre-SF(−)hCG (66 h). However, the nuclear maturation rate did not vary between the regular and Pre-SF(−)hCG culture systems. Thus, culturing small antral follicle-derived oocytes in Pre-SF(+)hCG for 59 h, which provides a favorable environment and promotes in the capacity of porcine oocytes, may increase their nuclear maturation rate.

To get a better understanding of an increased nuclear maturation of oocytes, the culture conditions during oocyte maturation strongly affect the cumulus expansion, meiotic maturation, and developmental competence of embryos. Changes in the cumulus expansion and nuclear maturation of porcine COCs are directly associated with the developmental competence of oocytes [19]. The cytoplasmic maturation and consequent developmental capability of mammalian oocytes require bidirectional communication between cumulus cells and oocytes [20]. To reveal the optimal culture conditions, the expression levels of genes involved in regulation of cumulus expansion during oocyte maturation (*HAS2*, *PTGS2*, *TNFAIP6*, and *PTX3*) under pre-IVM culture systems were compared to those in the regular culture system. Before maturation, the expression levels of genes regulating the cumulus expansion were higher in the oocytes derived from the medium antral follicles than those derived from small antral follicles. After maturation, the oocytes derived from both follicles exhibited significant increases in the levels of all genes, with the exception of *PTX3*, which exhibited a tendency toward significance, compared with the levels in control cells. The oocyte actively participates in cumulus expansion by secreting growth differentiation factor 9 (GDF9) to promote expansion. GDF9 increases the formation of hyaluronic acid via inducing *HAS2* in cumulus cells, which leads to cumulus expansion [20]. Hyaluronic acid, a product of *HAS2*, is a crucial part of the matrix which develops during cumulus expansion in response to the ovulatory luteinizing hormone surge which is also essential for cumulus expansion. Furthermore, the oocyte-derived GDF9 controls the expression of *HAS2*, and it was found that *HAS2* expression was greater in the cumulus cells of mature oocytes. Additionally, GDF9 promotes cumulus expansion by inducing *PTGS2* and the subsequent production of prostaglandin E2 (*PGE2*). Both of *PTGS2* and *PGE2* contribute to the expansion of cumulus cells in cattle [21]. In mice, it has been reported that ovulation, fertilization, decidualization, and implantation are defective due to lack of functional *PTGS2* [22]. An investigation showed that disruption of *TNFAIP6* could cause severe infertility because of defective cumulus expansion and a fault in its organization, which strengthens the idea that optimal expansion of the cumulus mass is crucial for ovulation in mice [23,24]. The mucoelastic extracellular matrix is stabilized by the presence of *TNFAIP6* protein in porcine COCs. Cumulus cells from competent oocytes in cattle had considerably more *TNFAIP6* mRNA than those from incompetent oocytes [25]. Cumulus expansion is significantly assisted by *PTX3* which localizes in the cumulus matrix. In humans, the cumulus cells collected from mature oocytes which were able to develop to the 8-cell stage on day 3 after fertilization were discovered to express higher *PTX3*. It was noticed that some of the cumulus cells appeared to have 12-fold increase in *PTX3* expression. In addition, those embryos derived from oocytes with higher *PTX3* expression in the expanded cumulus cells had higher implantation rate after embryo transfer [26]. These findings point out that pre-IVM culture systems upregulate the expression levels of cumulus expansion–related genes in oocytes derived from small antral follicles. Furthermore, the upregulation of these genes can be correlated to the morphological and physiological characteristics of mature oocytes and may provide a novel approach to predicting a successful IVM culture system.

Although we successfully established the IVM condition to enhance the nuclear maturation rate of oocytes derived from small antral follicles, the developmental competence of these oocytes is still lower compared to those produced in the regular culture systems. In the present study, we improved the quality and development of blastocyst formation by supplementing with melatonin during IVC period. After melatonin supplementation during IVC, no differences were observed in cleavage rates of the oocytes cultured in the regular and pre-IVM culture systems. However, the blastocyst formation rate and total cell number increased on Day 7 after PA. Moreover, melatonin supplementation improved the quality and developmental competence of the porcine oocytes. Blastocyst formation is a key indicator of developmental competence and optimal culture conditions; the total number of cells in a blastocyst is a standard indicator of blastocyst quality [27]. Melatonin supplementation assists in enhancing the quality and developmental potential of porcine *in vitro* fertilized embryos [12], and enhances the developing capacity of *in vitro* PA porcine embryos [28]. Also, it has been observed that melatonin enhanced damage repair during somatic cell reprogramming following H_2_O_2_-induced oxidative stress, and promotion of the development of porcine somatic cell nuclear transfer (SCNT) embryos, indicating that melatonin protects against oxidative stress during SCNT embryo development. Free radical generation is excessive under situations of oxidative stress, which may disrupt the equilibrium between ROS and antioxidants. Porcine embryos are very vulnerable to ROS-caused damage [29]. Thus, using antioxidants to reduce excessive ROS generation may help pig embryo growth more successfully. *In vitro* experiments on pig embryos also showed that exogenous melatonin might decrease ROS formation, increase GSH synthesis, and prevent apoptosis [30]. In a relevant study, the levels of intracellular ROS and DNA damage were higher in oocytes cultured for 52 h than in those cultured for 44 h [31]. The supplementation of exogenous melatonin may reduce intracellular ROS levels, increase intracellular GSH levels, and inhibit apoptosis in porcine embryos [13]. Melatonin obviously supports the development of pig embryos by enhancing blastocyst formation and increases the total cell number in embryos. Melatonin has also been demonstrated to be essential for pre-implantation development and implantation of embryos [32]. Furthermore, changing the culture medium to fresh IVC medium may reduce the level of free oxygen radicals or ROS, which are toxic products generated during pig embryo culture, in the cell membrane and DNA [33]. These results suggest that melatonin supplementation during IVC improves the quality and development of porcine embryos by increasing the total number of blastocyst cells and the rate at which blastocyst form after PA.

In the current study, the intracellular ROS levels in the oocytes increased after induction using the pre-IVM culture system, whereas the intracellular GSH levels decreased. This is consistent with previous research suggesting that although the pre-IVM culture system could improve the nuclear maturation of oocytes derived from small antral follicles, it had other critical effects, for instance, on ROS generation, DNA damage, and apoptosis in subsequent porcine embryos [31]. Melatonin has been reported to increase maturation and subsequent fertilization rates in human oocytes cultured *in vitro* by reducing oxidative stress [34]. Melatonin supplementation of the maturation medium in bovine decreased the production of ROS, DNA damage, and apoptosis [35,36]. Oxidative stress could be triggered by the accumulating ROS levels in oocytes, which can damage proteins and nucleic acids [37]. To alleviate the aforementioned negative effects, we added exogenous melatonin to the pre-IVM culture system during the IVM period (37 h). Melatonin supplementation exerted no prominent effect on the nuclear maturation rate. Nevertheless, it reduced the level of intracellular ROS and increased that of intracellular GSH in the oocytes, thus improving the ooplasmic maturation and developmental competence [38]. Melatonin works by activating antioxidant enzymes to detoxify reactive oxygen. Melatonin reduces ROS formation in pigs, which promotes the development of poor-quality oocytes when combined with prolonged IVM [31]. Melatonin supplementation improves the IVM of oocytes under heat stress by elevating intracellular GSH and ATP levels and diminishing ROS levels in porcine oocytes [39], and it improves oocyte quality during IVM by upregulating antioxidant gene expression and intracellular GSH and ATP levels in cattle [40]. Therefore, melatonin supplementation has the potential to protect oocytes from the negative impacts of intracellular ROS during IVM.

## CONCLUSION

This study provides new information on the developmental competence of oocytes derived from small antral follicles and improves embryonic development for those oocytes by supplementing with melatonin. The Pre-SF(+)hCG culture system could improve the nuclear maturation rate of porcine oocytes derived from small antral follicles and increase the expression of genes associated with cumulus cell expansion. However, although the Pre-SF(+)hCG culture system can improve nuclear maturation, the blastocyst formation rate found in these oocytes remains lower than those from the regular culture system. Therefore, the addition of melatonin during IVC can improve the quality and developmental capacity of oocytes derived from the Pre-SF(+)hCG culture system (particularly the total cell number and blastocyst formation rate). Furthermore, the melatonin supplementation during the IVM period has a positive influence by reducing and increasing intracellular ROS and GSH levels in the oocytes, respectively. Therefore, addition of melatonin in IVM medium would benefit the subsequent porcine embryonic development.

## Figures and Tables

**Table 1:** Primers used for real-time polymerase chain reaction

Genes	Primer sequences (5′–3′)		Product size (bp)	Accession No.
	Forward	Reverse		
*GAPDH*	GGGCATGAACCATGAGAAGT	AAGCAGGGATGATGTTCTGG	230	AF017079
*HAS2*	ATGACAGGCATCTAACGAACC	ACATCTTGGCGGGAAGTAAA	420	AB050389
*PTGS2*	ACATCTGATTGATAGCCCACC	CCTCGCTTCTGATCTGTCTTG	288	NM_001244783
*TNFAIP6*	CAGTTAGAGGCAGCCAGAAAA	CAACATAGTCAGCCAAGCAAG	373	NM_001159607
*PTX3*	CGCCAATACTGTGATTTCCC	CCAGATATTGAAGCCTGTGAGTC	247	NM_001244783

*GAPDH*, glyceraldehyde 3-phosphate dehydrogenase; *HAS2*, hyaluronan synthase 2; *PTGS2*, prostaglandin-endoperoxide synthase 2; *TNFAIP6*, tumor necrosis factor–stimulated gene 6; *PTX3*, pentraxin 3.
